# Alterations in the stomatognathic system due to amyotrophic lateral sclerosis

**DOI:** 10.1590/1678-7757-2017-0408

**Published:** 2018-05-22

**Authors:** Lígia Maria Napolitano Gonçalves, Marcelo Palinkas, Jaime Eduardo Cecilio Hallak, Wilson Marques, Paulo Batista de Vasconcelos, Nicolly Parente Ribeiro Frota, Isabela Hallak Regalo, Selma Siéssere, Simone Cecilio Hallak Regalo

**Affiliations:** 1 Universidade de São Paulo, Faculdade de Odontologia de Ribeirão Preto, Departamento de Morfologia, Fisiologia e Patologia Básica, Ribeirão Preto, São Paulo, Brasil.; 2 Faculdade Anhanguera de Ribeirão Preto, São Paulo, Brasil.; 3 Universidade de São Paulo, Faculdade de Medicina de Ribeirão Preto, Departamento de Neuropsiquiatria e Psicologia Médica, Ribeirão Preto, São Paulo, Brasil.

**Keywords:** Amyotrophic lateral sclerosis, Masticatory muscles, Electromyography, Bite force, Ultrasound

## Abstract

**Objectives:**

To compare the molar bite force, electromyographic activity, chewing efficiency and thickness of the masseter and temporalis muscles in individuals with amyotrophic lateral sclerosis (ALS) and healthy individuals.

**Material and Methods:**

Thirty individuals enrolled in the study were divided into the study group (with ALS, n=15) and control group (healthy individuals, n=15). Data regarding molar bite force (right and left), electromyographic activity (mandibular rest, right and left laterality, protrusion, and maximum voluntary contraction), chewing efficiency (habitual and non-habitual), and masticatory muscle thickness (rest and maximum voluntary contraction) were tabulated and subjected to statistical analysis (Student’s t-test, p≤0.05).

**Results:**

Comparisons between the groups demonstrated a statistically significant increase in the electromyographic activity of the right masseter (p=0.03) and left masseter (p=0.03) muscles during mandibular rest; left masseter (p=0.00), right temporalis (p=0.00), and left temporalis (p=0.03) muscles during protrusion; and right masseter (p=0.00), left masseter (p=0.00), and left temporalis (p=0.00) muscles during left laterality, in individuals with ALS as compared with healthy individuals. A statistically significant decrease was observed in the habitual chewing efficiency of the right masseter (p=0.00) and right temporalis (p=0.04) muscles in individuals with ALS. No statistically significant difference between the groups was found the masticatory muscle thickness and maximal molar bite force.

**Conclusions:**

ALS may lead to modifications in the activities of the stomatognathic system, including muscular hyperactivity and reduction in chewing efficiency; however, no change has been observed in the masticatory muscle thickness and molar bite force.

## Introduction

Amyotrophic lateral sclerosis (ALS) is a chronic, progressive, complex, age-associated syndrome that affects the functions of the superior and inferior neuromotor system through sclerosis and destruction of cells and nerves, leading to the patient’s weakness, causing progressive paralysis, and eventually resulting in death^20^. ALS may be classified as sporadic, which is the most common form globally, and familial, which affects individuals by autosomal dominant inheritance^2^. In the literature, few studies on the effect of ALS on masticatory muscles can be found, but there is a possibility of disease interference in mandibular mobility and orofacial pain^25^.

Muscular degeneration begins at the body extremities. It is usually asymmetrical and presents bilateral progression, thereby resulting in the individual’s inability to perform voluntary movements during breathing, swallowing, and phonation; it does not alter the sensory and intellectual functioning^9^.

The global incidence proportion of ALS is 1.9 to 4.5/100,000 persons *per* year^1^. As age is an important predisposing factor, its prevalence increases to 6/100,000 people, among individuals between 58-60 years^29^.

Individuals with ALS present with a compromised skeletal muscle system led research groups to assess the consequences of the disease in the human body^13^ ^,^ ^15^. ASL promotes functional changes in the stomatognathic system, such as dysphonia, dysarthria, dysphagia, salivation and pattern of force and tongue activity^27^. However, gaps still exist in our knowledge of the impact of ALS on the stomatognathic system, especially regarding masseter and temporal, which are important muscles for mastication. Therefore, the objective of this clinical research was to demonstrate possible changes in the activity of the stomatognathic system in individuals with ALS by assessing the maximal molar bite force, electromyographic activity, chewing efficiency, and thickness of the masseter and temporalis muscles.

## Material and methods

### Study population

The study was approved by the Research Ethics Committee (protocol #13071913.3.3001.5419), under Resolution 466/12 of the National Health Council. All procedures performed in this study with human participants were in accordance with the ethical standards of the institutional and national research committees and with the Declaration of Helsinki of 1975, revised in 2000.

Seventy individuals with ALS, aged 18-68 years, without severe muscle impairment and unilateral atrophy were assessed and selected at the Department of Neurosciences and Behavioral Sciences, Ribeirão Preto, São Paulo, Brazil. The individuals with ALS had their diagnosis validated by neurologists, through clinical examination and diagnostic tests, such as electroneuromyography, nerve conduction velocity, blood and urine tests, and neurological examination. All subjects were in the early stage of disease. All subjects with ALS received the same medication (Riluzole) and were instructed to avoid caffeine, anti-inflammatory, tranquillizer, vasodilator and antidepressants, to prevent the reduction of drug potentiation^19^.

The exclusion criteria were cognitive changes (n=2); necessity for ventilatory support (n=6); diseases of the anterior medullary horn (n=9); dementia (n=3); visual, autonomic, and sphincter disorders (n=8); palatal and mandibular tori (n=5); temporomandibular disorders (n=7); absence of first permanent molars (n=8); and moderate and severe malocclusion (n=7). Therefore, 15 individuals with ALS were recruited and participated in the study (mean ± SD, 42.13±4.09, amyotrophic lateral sclerosis group – ALSG). Fifteen healthy individuals with Angle Class I occlusion (mean ± SD, 43.33±3.93, without the disease group – CG) were matched for age, sex, and anthropometric measurements with the participants in the ALSG (Table 1).

**Table 1:** Comparison of the mean age and anthropometric measurement according to the groups. ALSG: Amyotrophic lateral sclerosis group; CG: control group. t test (p≤0.05)

Anthropometric Measurement	ALSG	CG	p-value
Age (years)	42.13±4.09	43.33±3.93	0.83
Weight (Kg)	73.46±4.44	71.09±3.05	0.66
Height (cm)	1.63±0.03	1.67±0.03	0.51

As the study sample was 15 individuals with ALS, the standard deviation of responses was increased to enhance the significance of effects observed. A maximum level of significance α=0.05 and a minimal test power of 95% were adopted. A difference of twice the standard deviation is considered significant. In this study, the greatest difference from the standard deviation was applied for calculations.

The evaluation of electromyographic, masticatory efficiency, thickness and bite force records were performed by a single trained professional. Intra-examiner calibration was performed for all analyzes of this study. The reliability of the intra-rater was good by calculating the intra-class coefficient (ICC). Reliability was considered acceptable for muscle thickness (ICC=0.999), electromyographic activity (ICC=0.936) and maximal molar bite force (ICC=0.928).

### Electromyographic analysis

Electromyograph MyoSystem-BrI (DataHominis, Uberlândia, Minas Gerais, Brazil) was used to analyze the electromyographic activity of the right and left masseter, and right and left temporalis muscles. Specific dental clenching maneuvers were performed in maximum voluntary contraction, accompanied by palpation, for correct fixation of the electrodes^11^.

Electromyographic activity was evaluated during mandibular rest (4 s), dental clenching in maximum voluntary contraction with (4 s) and without inert material (4 s), right and left laterality (10 s), and protrusion (10 s). The inert material consisted of a folded paraffin sheet (Parafilm M^®^, Pechinery Plastic Packaging, Batavia, IL, USA; 18×17×4 mm; weight 245 mg) inserted between the occlusal surfaces of the first and second molars, on the right and left side.

### Masticatory efficiency analysis

The linear envelope of the electromyographic signals of the masticatory cycle was used to evaluate the efficiency of the right and left masseter, and the right and left temporalis muscles during non-habitual chewing with Parafilm M^®^ and habitual chewing with consistent (peanuts) and soft (raisins) food. The food belonged to the same batch and the amount dispensed was 5 g of each type of food, which was stored in individual plastic containers in a cool and ventilated place. The values of the integral of the linear envelope were calculated by evaluating the central cycles, eliminating the first cycle at the beginning of the masticatory process as it presents considerable variation in the mandibular movement pattern^23^.

### Muscle thickness analysis

NanoMaxx Ultrasound System equipment (SonoSite, Inc., Bothell, Washington, USA) was used to analyze the thickness (centimeters) of the right and left masseter, and right and left temporalis muscles. Digital palpation and linear transducer movement confirmed the location of masticatory muscles^3^. The head was positioned with the Frankfurt horizontal plane parallel to the ground^14^. Three ultrasonographic images were obtained during mandibular rest and dental clenching in maximal voluntary contraction, with a 2-minute interval between measurements. The averages of the values obtained from these three ultrasound images were calculated in each clinical condition, making a total of six.

### Maximal molar bite force analysis

The maximal molar bite force (right and left) data were obtained using a digital dynamometer model IDDK (Kratos, Cotia, São Paulo, Brazil), with a capacity up to 980.66 N. Three measurements were made in the region of the right and left first molars^24^. The individual bit of the device consists of two stems protected with latex finger cots for biosafety. Maximum effort was applied, alternating between right and left sides, with a 2-minute rest period between each record, to minimize possible interferences and erroneous results^7^. The maximal molar bite force on both sides was recorded.

### Statistical analysis

Electromyographic data obtained during dental clenching in maximal voluntary contraction with Parafilm M^®^, were normalized. The analyses were performed using SPSS version 21.0 for Windows (SPSS Inc., Chicago, IL, USA). Descriptive analysis (means and standard error) and Shapiro-Wilk normality test were performed for each variable. The values were compared using independent samples t-test (p≤0.05).

## Results

Table 1 shows the average age and anthropometric measures for the ALSG and CG. No statistically significant differences were found between the groups.

**Figure 1:**
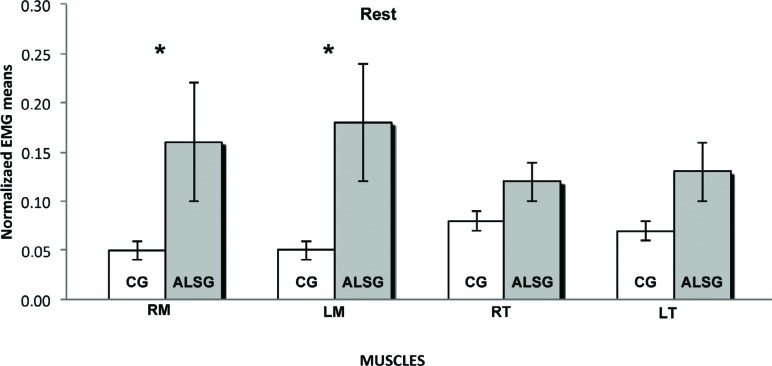
Normalized electromyography data of masseter (RM: right masseter; LM: left masseter) and temporalis (RT: right temporal; LT: left temporal) muscles for CG and ALSG in the rest. *statistically significant difference (p≤0.05)

**Figure 2:**
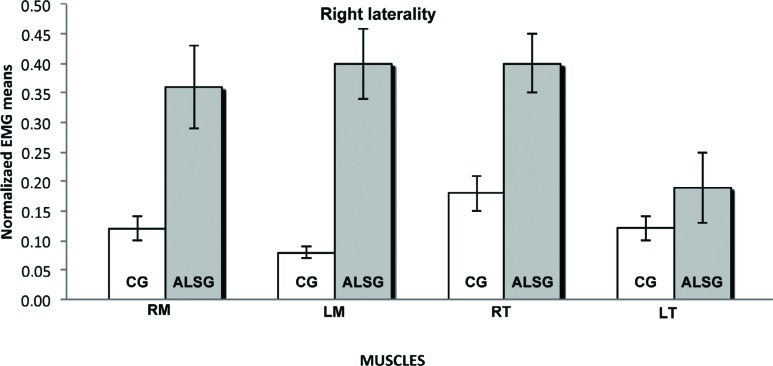
Normalized electromyography data of masseter (RM: right masseter; LM: left masseter) and temporalis (RT: right temporal; LT: left temporal) muscles for CG and ALSG in the right laterality

**Figure 3:**
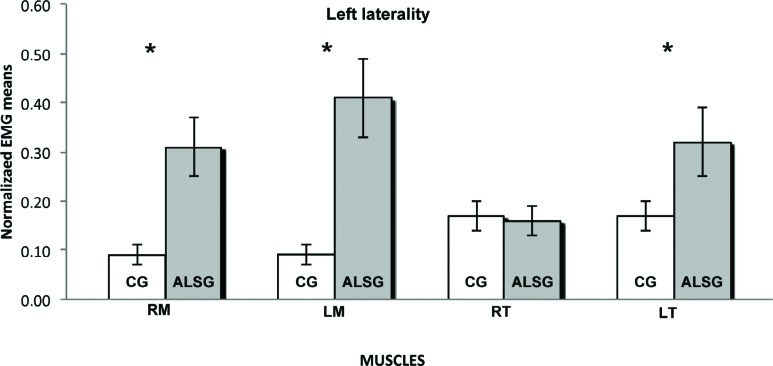
Normalized electromyography data of masseter (RM: right masseter; LM: left masseter) and temporalis (RT: right temporal; LT: left temporal) muscles for CG and ALSG in the left laterality. *statistically significant difference (p≤0.05)

**Figure 4:**
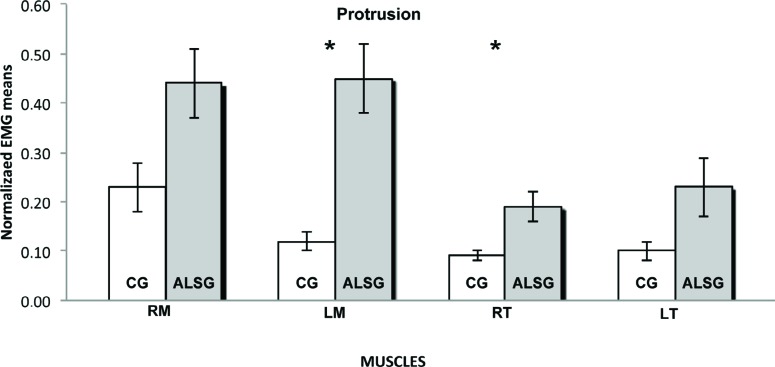
Normalized electromyography data of masseter (RM: right masseter; LM: left masseter) and temporalis (RT: right temporal; LT: left temporal) muscles for CG and ALSG in the protrusion. *statistically significant difference (p≤0.05)

**Figure 5:**
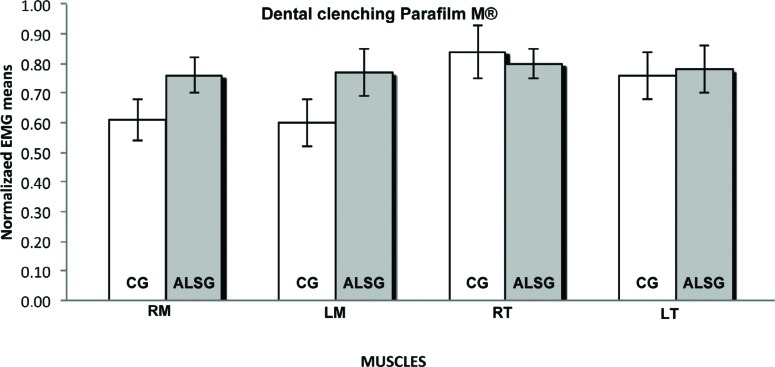
Normalized electromyography data of masseter (RM: right masseter; LM: left masseter) and temporalis (RT: right temporal; LT: left temporal) muscles for CG and ALSG in the dental clenching Parafilm M^®^

Table 2 shows the normalized electromyographic data at each clinical condition for the ALSG and CG. Statistically significant differences (p≤0.05) were found in the electromyographic activity between the ALSG and CG during mandibular rest to right masseter (p=0.03) and left masseter (p=0.03) muscles; protrusion to left masseter (p=0.001), right temporal (p=0.001) and left temporal (p=0.03) muscles; and left laterality to right masseter (p=0.001), left masseter (p=0.001) and left temporal (p=0.002) muscles (Figures 1-5).

**Table 2:** Comparison of the mean normalized electromyographic data during mandibular task according to the groups. ALSG: Amyotrophic lateral sclerosis group; CG: control group; SD: standard deviation; RM: right masseter; LM: left masseter; RT: right temporal; LT: left temporal. Statistical significance was calculated using t test (p≤0.05). Significant p-values are indicated in bold

Mandibular task	Muscles	ALSG	CG	p-value
Rest	RM	0.16±0.06	0.05±0.01	**0.03**
	LM	0.18±0.06	0.05±0.01	**0.03**
	RT	0.12±0.02	0.08±0.01	0.19
	LT	0.13±0.03	0.07±0.01	0.08
Right laterality	RM	0.36±0.07	0.12±0.02	0.14
	LM	0.40±0.06	0.08±0.01	0.41
	RT	0.34±0.05	0.18±0.03	0.61
	LT	0.19±0.05	0.12±0.02	0.74
Left laterality	RM	0.31±0.06	0.09±0.02	0.001
	LM	0.41±0.08	0.09±0.02	0.001
	RT	0.16±0.03	0.17±0.03	0.08
	LT	0.32±0.07	0.17±0.03	0.002
Protrusion	RM	0.44±0.07	0.23±0.05	0.16
	LM	0.45±0.07	0.12±0.02	0.001
	RT	0.19±0.03	0.09±0.01	0.001
	LT	0.23±0.06	0.10±0.02	**0.03**
Dental clenching Parafilm M^®^	RM	0.76±0.06	0.61±0.07	0.43
	LM	0.77±0.08	0.60±0.08	0.88
	RT	0.80±0.05	0.84±0.09	0.68
	LT	0.78±0.08	0.76±0.08	0.60

**Figure 6:**
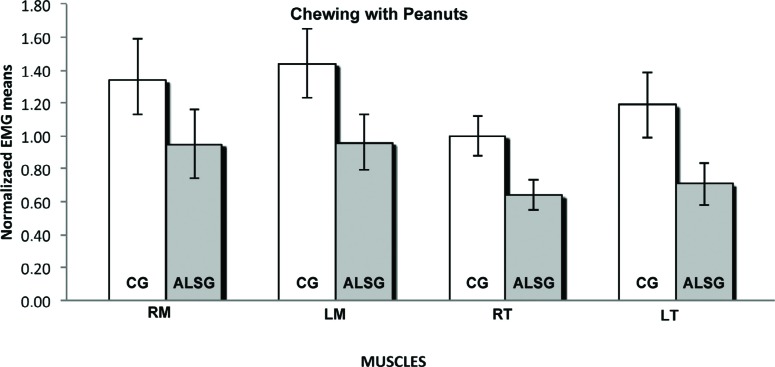
Normalized electromyography data of masseter (RM: right masseter; LM: left masseter) and temporalis (RT: right temporal; LT: left temporal) muscles for CG and ALSG in the chewing with peanuts

**Figure 7:**
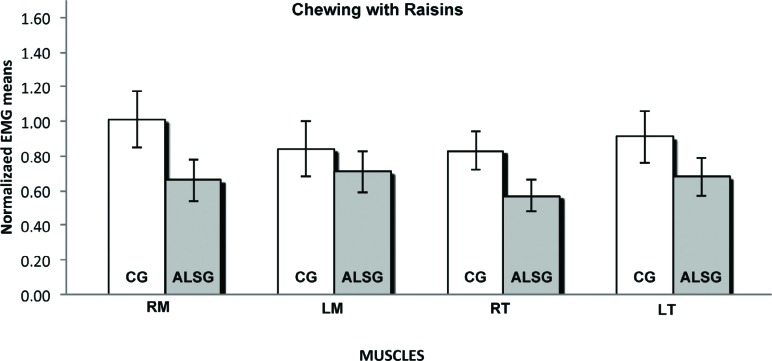
Normalized electromyography data of masseter (RM: right masseter; LM: left masseter) and temporalis (RT: right temporal; LT: left temporal) muscles for CG and ALSG in the chewing with raisins

**Figure 8:**
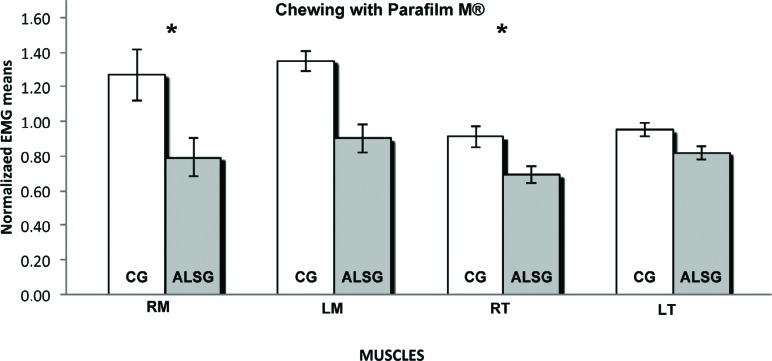
Normalized electromyography data of masseter (RM: right masseter; LM: left masseter) and temporalis (RT: right temporal; LT: left temporal) muscles for CG and ALSG in the chewing with Parafilm M^®^. *statistically significant difference (p≤0.05)

Table 3 presents the averages of the habitual and non-habitual chewing efficiency for the ALSG and CG. A statistically significant difference (p≤0.05) between the ALSG and CG regarding non-habitual chewing (right masseter and right temporalis muscles) was observed (Figures 6-8).

**Table 3:** Comparison of the mean habitual and non-habitual chewing data according to the groups. ALSG: Amyotrophic lateral sclerosis group; CG: control group; SD: standard deviation; RM: right masseter; LM: left masseter; RT: right temporal; LT: left temporal. Statistical significance was calculated using t test (p≤0.05). Significant p-values are indicated in bold

Chewing	Muscles	ALSG	CG	p-value
Peanuts	RM	0.95±0.21	1.34±0.25	0.50
	LM	0.96±0.17	1.44±0.21	0.24
	RT	0.64±0.09	1.00±0.12	0.28
	LT	0.71±0.13	1.19±0.20	0.18
Raisins	RM	0.66±0.12	1.01±0.16	0.84
	LM	0.71±0.12	0.84±0.16	0.64
	RT	0.57±0.09	0.83±0.11	0.30
	LT	0.68±0.11	0.91±0.15	0.12
Parafilm M^®^	RM	0.79±0.08	1.27±0.06	**0.00**
	LM	0.90±0.05	1.35±0.06	0.06
	RT	0.69±0.04	0.91±0.04	**0.04**
	LT	0.82±0.04	0.95±0.04	0.91

**Figure 9:**
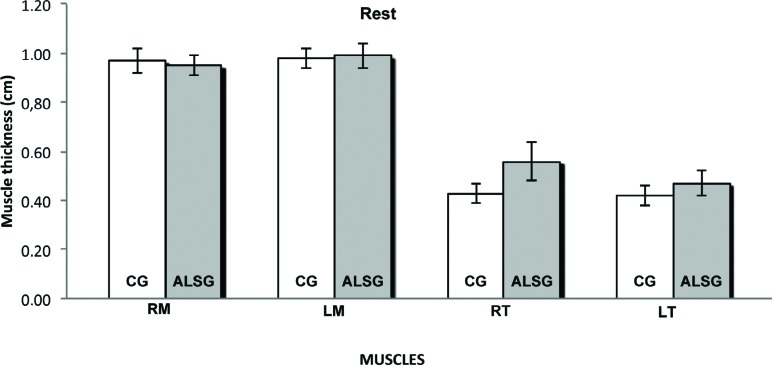
Masticatory muscle’ thickness data of masseter (RM: right masseter; LM: left masseter) and temporalis (RT: right temporal; LT: left temporal) muscles for CG and ALSG in the rest

**Figure 10:**
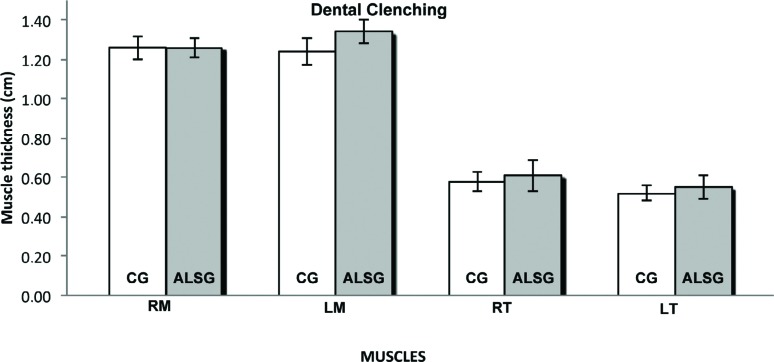
Masticatory muscle thickness data of masseter (RM: right masseter; LM: left masseter) and temporalis (RT: right temporal; LT: left temporal) muscles for CG and ALSG in the dental clenching

Table 4 shows the averages of the thickness of the right and left masseter, and right and left temporalis muscles during maximal voluntary contraction and mandibular rest for the ALSG and CG. No statistically significant differences were found between the groups (Figures 9, 10).

**Table 4:** Comparison of the mean masticatory muscle thickness (cm) during mandibular task according to the groups. ALSG: Amyotrophic lateral sclerosis group; CG: control group; SD: standard deviation; RM: right masseter; LM: left masseter; RT: right temporal; LT: left temporal. Statistical significance was calculated using t test (p≤0.05). Significant p-values are indicated in bold

Mandibular task and Muscles	ALSG	CG	p-value
Rest			
RM	0.95±0.04	0.97±0.05	0.63
LM	0.99±0.05	0.98±0.04	0.70
RT	0.56±0.08	0.43±0.04	0.10
LT	0.47±0.05	0.42±0.04	0.11
Dental Clenching			
RM	1.26±0.05	1.26±0.06	0.43
LM	1.34±0.06	1.24±0.07	0.41
RT	0.61±0.08	0.58±0.05	0.14
LT	0.55±0.06	0.52±0.04	0.31

**Figure 11:**
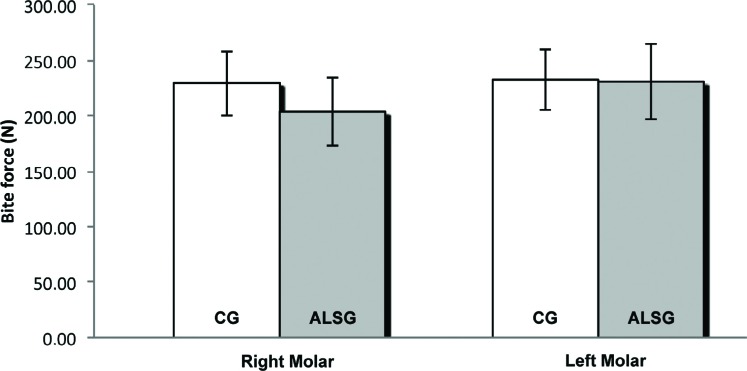
Maximum molar bite force data of CG and ALSG (right molar and left molar)

The average values of the maximal molar bite force (right and left sides) are presented in Table 5. No statistically significant differences were found between the ALSG and CG (Figure 11).

**Table 5:** Comparison of the mean bite force (N) according to the groups. ALSG: Amyotrophic lateral sclerosis group; CG: control group; SD: standard deviation; RM: right masseter; LM: left masseter; RT: right temporal; LT: left temporal. Statistical significance was calculated using t test (p≤0.05)

Bite Force	ALSG	CG	p-value
Right Molar	203.68±31.08	229.68±29.32	0.553
Left Molar	230.75±34.22	232.41±27.65	0.970

## Discussion

Our results demonstrated the possibility of functional alterations in the stomatognathic system of individuals with ALS. We have used different internationally standardized techniques to evaluate the masseter and temporalis muscles accurately. Masseter and temporal are masticatory muscles. The localization and morphology of these muscles allow the analysis by EMG. The advantages of their use are demonstrated by the ease access and the specific function of these two muscles. The obtained results of increased muscle activity during rest and decreased chewing efficiency could be perceptible signs of the early stages of ALS.

The electromyographic data of the masseter and temporalis muscles presented abnormalities during mandibular rest in individuals with ALS. This was an indication of absence of the contraction mechanism of motor units^6^. Mandibular balance is maintained by viscoelasticity of the masticatory muscles; proprioceptive action of the ligaments, tendons, joint capsule; and atmospheric pressure^22^. We observed that ALSG presented greater electromyographic activity regarding the masticatory muscles, during mandibular rest. These results are in accordance with earlier results in scientific literature wherein the presence of electrical hyperactivity in the skeletal striated muscles has been correlated to situations of continuous stress and/or the presence of muscular dysfunctions^10^. Higher activity was also observed for masseter and temporal muscles in subject with Duchenne muscular dystrophy^13^. Individuals with ALS present with continual stress due to emotional problems related to the neurodegenerative disease^4^. Our research did not evaluate the levels of stress in individuals with ALS.

Our results demonstrated greater muscular hyperactivity in the ALSG when compared with CG. Instability in the postural biomechanics of individuals with neurodegenerative diseases needs greater physical effort, requiring a greater recruitment of muscle fibers when compared with the physical effort and recruitment of muscle fibers in healthy individuals. This is the mechanism for muscular hyperactivity in individuals with neurodegenerative diseases. We have also demonstrated that the masseter muscles are more active when compared with the temporalis muscles, in protrusion. Results agree with the normality patterns in the movements in this mandibular task^13^.

Regarding laterality (right and left) of the mandible, the normalized electromyographic activity of the masticatory muscles was higher in the ALSG when compared with CG. The same was not observed in subjects with sleep bruxism^22^. It was evident that our result did not follow the normality pattern of neuroanatomical muscle activation, in which larger electromyographic records were expected in the temporalis muscle of the ipsilateral side of the mandible; as for the masseter muscle, the greatest activity expected would be the contralateral^10^, and it was not verified in the execution of this clinical condition in our study.

The normalized electromyographic activity of the masseter muscles when compared with the electromyographic activity of the temporalis muscles is lower, during maximum voluntary contraction with Parafilm M^®^. These data are in accordance with those in the scientific literature, verifying the lower myoelectric activity pattern in the masseter muscle when compared with myoelectric activity in the temporalis muscle^15^. This relationship is explained by the activation of the masseter muscle for mandibular support, with morphological characteristics related to strength and power; however, everyday stress factors can alter this function completely, overloading the temporalis muscle, with characteristics related to adjustment and synchronization of the mandibular dynamics^26^.

In ALS, muscle spasms are continuous and do not alter during voluntary movements^17^. These rapid, spontaneous, and intermittent contractions of muscle fibers are called fasciculation^4^. They demonstrate the potential of abnormally functioning motor units in causing hyperactivity of the cardiac and skeletal muscle^30^.

In this study, electromyography was performed in a humane manner in individuals with ALS to evaluate masticatory function. They were provided the option of spitting out the sample test foods after performing the habitual chewing movements with soft food (raisins) and consistent food (peanuts).

The measurement of chewing efficiency was very complex; however, the use of recognized standard techniques enabled the reproduction of the results in a reliable manner^28^. Moreover, we used the electromyographic signal values of the integral of the linear envelope of the masticatory cycles to verify the functional alteration in chewing efficiency produced by ALS. Our results showed lower normalized electromyographic averages of masticatory cycles regarding the masseter and temporalis muscles, in the ALSG when compared with those in the CG; these results were observed in both habitual and non-habitual chewing. Palinkas, et al.^22^ (2016) also observed lower masticatory efficiency in subjects with sleep bruxism, with lower electromyographic averages for right masseter and left temporal during habitual chewing. Non-habitual chewing, characterized by short dynamic excursion with buccal opening and hinge motion of the temporomandibular joint, was requested to diminish the effects of changes in length and tension of the muscle^12^.

During non-habitual (Parafilm M^®^) chewing, statistically significant functional changes were observed in the right masseter and right temporalis muscles, between the ALSG and CG. Individuals with morphofunctional changes are believed to present a greater recruitment of muscle fibers to perform the same masticatory functions as healthy individuals, resulting in greater physical effort and decrease in masticatory efficiency^18^.

Similarly, muscle thickness can also interfere with the masticatory process, and normality patterns related to the thickness of the masticatory muscles are well delineated^4^. In this study, the thickness of the masseter and temporalis muscles during mandibular rest and dental clenching in maximum voluntary contraction were well defined, allowing precise measurements, without statistically significant differences in the averages for the analyzed muscles, between the ALSG and CG. Based on the results of the study by Arts, et al.^5^ (2008), muscle thickness is lower in individuals with ALS; however, the muscles analyzed in their study were not the muscles of the stomatognathic system.

Our results led to some questions regarding the thickness of the masseter and temporalis muscles. Can ALS occur without severe muscle impairment atrophying the skeletal striated musculature? Can the function of masticatory muscles be protected from the disease, in such a way that there is no change in muscle thickness? Further studies will be needed to answer these questions.

The evaluation of maximal molar bite force is fundamental to investigate the impairment of masticatory efficiency because of the pre-established theory that the better the system, the greater the force exerted^16^ ^,^ ^22^. Our results demonstrated that no significant clinical differences were found between ALSG and CG regarding maximal molar bite force, for both the right and left sides. These results are in agreement with the results of the study by Ohnuki, et al.^21^ (2002), who stated that the occlusal strength of individuals with motor neuron diseases, including ALS, did not change when compared with the occlusal strength of healthy individuals.

To complement the results of this research, the non-correlation between age and sex and the results was statistically affirmed.

Although all the subjects were in the early stage of the disease, they did not present the same symptoms, thus this condition may be a limitation of our study. Therefore, the static and dynamic evaluations of the stomatognathic system are of paramount importance to the mechanism of ALS and role of adaptive muscular remodeling which exists in situations where physical stimuli are constant. Therefore, this may produce quantitative and qualitative changes in the structure of the masticatory muscles^8^.

Our study demonstrates the morphological and functional evaluation of the stomatognathic system in individuals with ALS, offering important contributions to our knowledge in the field of health. All health professionals, especially those in the Dentistry field, should carefully plan and apply adequate therapies to avoid greater damages to muscular system in individuals with ALS and the monitoring of these individuals should be effective and constant. Other studies should be performed in subjects with ALS in more advanced stages, allowing a better understanding of the performance of the masticatory muscles.

## Conclusions

This clinical study demonstrates the possibility of altered activity of the stomatognathic system in individuals with ALS, including an increase in electromyographic activity of the masseter and temporalis muscles and a reduction in masticatory efficiency; signs of the early stages of ALS may be perceptible. No changes were observed regarding masseter and temporalis muscle thickness and molar bite force.
